# *In silico* analysis of heme oxygenase structural homologues identifies group‐specific conservations

**DOI:** 10.1002/2211-5463.12275

**Published:** 2017-09-04

**Authors:** Joseph Irvin, Alexander J. Ropelewski, John Perozich

**Affiliations:** ^1^ Department of Biology Franciscan University of Steubenville OH USA; ^2^ Pittsburgh Supercomputing Center Carnegie Mellon University Pittsburgh PA USA

**Keywords:** group entropy, heme oxygenase, methane monooxygenase, ribonucleotide reductase, thiaminase

## Abstract

Heme oxygenases (HO) catalyze the breakdown of heme, aiding the recycling of its components. Several other enzymes have homologous tertiary structures to HOs, while sharing little sequence homology. These homologues include thiaminases, the hydroxylase component of methane monooxygenases, and the R2 component of Class I ribonucleotide reductases (RNR). This study compared these structural homologues of HO, using a large number of protein sequences for each homologue. Alignment of a total of 472 sequences showed little sequence conservation, with no residues having conservation in more than 80% of aligned sequences and only five residues conserved in at least 60% of the sequences. Fourteen additional positions, most of which were critical for hydrophobic packing, displayed amino acid similarity of 60% or higher. Ten conserved sequence motifs were identified in HOs and RNRs. Phylogenetic analysis verified the existence of the four distinct groups of HO homologues, which were then analyzed by group entropy analysis to identify residues critical to the unique function of each enzyme. Other methods for determining functional residues were also performed. Several common index positions identified represent critical evolutionary changes that resulted in the unique function of each enzyme, suggesting potential targets for site‐directed mutagenesis. These positions included residues that coordinate ligands, form the active sites, and maintain enzyme structure.

**Enzymes:**

Heme oxygenase (EC 1.14.14.18), methane monooxygenase (EC 1.14.13.25), ribonucleotide reductase (EC 1.17.4.1), thiaminase II (EC 3.5.99.2).

**Abbreviations**
BsThiaminase: *Bacillus subtilis* TenA thiaminase
EcRNR1A: RNR beta chain from *Escherichia coli*
hHO1: human heme oxygenase 1
HO: heme oxygenase
MMOH: methane monooxygenase hydroxylase
MMO: methane monooxygenase
PfThiaminase: thiaminase from *Pyrococcus furiosus*
RNR1A: homologue of RNR beta chain from *E. coli*
RNR1B: homologue of RNR R2F from *Salmonella typhimurium*
RNR: ribonucleotide reductase
Treg: transcriptional regulator

Heme oxygenase (HO, EC 1.14.14.18) aids in recycling iron and porphyrin rings by catalyzing the decomposition of heme to biliverdin, CO, and free iron. The reaction requires three molecules of oxygen, NADPH, and cytochrome P450 reductase. In animals, biliverdin is converted to bilirubin, which acts as an antioxidant‐protecting neurons 1 and which is later combined with glucuronic acid and excreted 2. CO functions as a signaling molecule via guanylate cyclase, playing several roles in vasoregulation 3. The iron released is the main source of metal in regenerating heme and nonheme cofactors 4, 5. Two HO isoforms exist in vertebrates, heme oxygenase‐1 (HO1) and heme oxygenase‐2 (HO2). HO1 and HO2 are both membrane‐bound proteins and share 55% identity in humans 6. Human HO2 is expressed constitutively in the liver, brain, and testes. Human HO1 is an inducible protein found in most tissues, especially the spleen and liver 7, 8. HO1 is also expressed in cancer cells. HO1 has been associated with adhesion and signaling in cancer cells, enabling growth and spread of tumors 9. In addition, elevated HO1 levels in mice appear to correlate with insulin resistance, while HO1‐knockout mice were insusceptible to insulin resistance and exhibited lower levels of inflammation 10. A heme oxygenase‐3 enzyme has been identified in humans, but is an inferior catalyst for heme breakdown 8. In *Arabidopsis*, which possesses four HO genes, HO is essential for the synthesis of light‐sensing phytochrome chromophores 11, 12. *Corynebacterium diphtheriae* uses a HO homologue (HmuO), which shares 33% sequence identity to human HO1, to acquire iron for growth from its host's own heme molecules 13.

There are several different enzymes with diverse functions that share structural homology to HOs. One of these enzymes is another oxidoreductase, methane monooxygenase (MMO, EC 1.14.13.25). MMO is found in aerobic methanogens and catalyzes the NAD(P)H‐dependent conversion of methane and oxygen to methanol 14, although it can act upon slightly longer hydrocarbons 15. There are three components to the structure of a soluble MMO: a reductase, a regulatory B component, and a hydroxylase (MMOH). The structure of MMOH has three subunits (α, β, and γ) and a hydroxo‐bridged diiron cluster. This is found in the α subunit only near the hydrophobic pocket that is the active site and is positioned between helices αB, αC, αE, and αF. Interaction of the β subunit with the reductase component of MMO affects the redox potential of this diiron cluster in the α subunit 16. Both the α and β subunits of MMOH show homology (approximately 12% sequence identity) to HOs and the other homologues addressed here 17.

Ribonucleotide reductases (RNRs, EC 1.17.4.1) are catalysts in the process of forming deoxyribonucleotides from ribonucleotides. The four deoxyribonucleotides that are produced are important for DNA synthesis in living organisms. RNRs have been subdivided into three classes based upon the way they form a radical in the reaction mechanism. Class I RNRs have a diiron cluster, while Class II RNRs use adenosylcobalamin and Class III RNRs utilize a iron‐sulfur cluster and S‐adenosylmethionine. While members of the three classes share little sequence homology, there is a core tertiary structure common to each 18. However, it has been noted that Class I RNRs also share structural homology to HOs, thiaminases, and MMOHs 17. Class I RNR in *Escherichia coli* is made up of two dimeric proteins, R1 and R2, which differ in structure. R1's structure includes binding sites for substrates, allosteric sites (the specificity and activity sites), and critical cysteine residues. R2, which is a homologue of HOs, has a highly oxidized redox center that is formed by a diiron cluster and a critical tyrosyl radical on Tyr122 that activates the substrate ribose. The tyrosyl radical is needed for the protein to act as an enzyme 19. Like MMOH, it utilizes oxygen and a diiron cluster; however, RNRs reduce hydroxyls on ribose, while MMOH acts to form a hydroxyl on methane. Class I RNRs have been subdivided into two groups: Ia, which are represented by *E. coli* R2 subunits, and Ib, which are represented by the R2F subunit from RNR from *Salmonella typhimurium*. Ib RNRs (RNR1Bs) appear to be solely in bacteria, while Ia RNRs (RNR1As) are in both bacteria and eukaryotes. While sharing a similar structure to Ia, Ib differs functionally through a lack of allosteric regulation and the need for small redox protein produced from the same operon as the RNR 20.

Another related enzyme is the aminopyrimidine aminohydrolase (thiaminase II; EC 3.5.99.2) TenA protein, which acts as a thiamin (vitamin B_1_) salvage enzyme that catalyzes hydrolysis of 4‐amino‐5‐aminomethyl‐2‐methylpyrimidine. TenA modifies the aminopyrimidine to hydroxypyrimidine and is found throughout all three domains of life 21. The TenA gene product was initially identified as a transcriptional regulator (Treg) of exoenzyme secretion in *Bacillus subtilis* 22. However, the TenA homologue in *Pyrococcus furiosus* lacks appropriate surface charges and therefore may not participate with DNA interactions 17. *Pyrococcus furiosus* TenA only shares 14% sequence identity to human HO1 17. The *Saccharomyces cerevisiae* THI20 protein is actually a trifunctional enzyme which includes a C‐terminal tetrameric TenA‐like domain fused to an N‐terminal ThiD domain which catalyzes another step in thiamin metabolism 23. French *et al*. 23 also identified two structural subclasses of TenAs: one group similar to *B. subtilis* TenA with a conserved cysteine and the other similar to *P. furiosus* TenA with a conserved glutamate instead.

There has not been an extensive comparative study performed on HO and its homologues: thiaminases, MMOHs, and Class I RNRs. The goal of this research was to align a large number of HO protein sequences and those for these related homologues. We then attempted to identify the structural and possible functional roles of conserved residues and sequence motifs in these enzymes. Group entropy analysis and other methods indicated group‐specific conservations for each homologue, identifying key positions that may contribute to the unique function of each enzyme.

## Results and Discussion

### Structure and residue conservations

**Figure 1:**
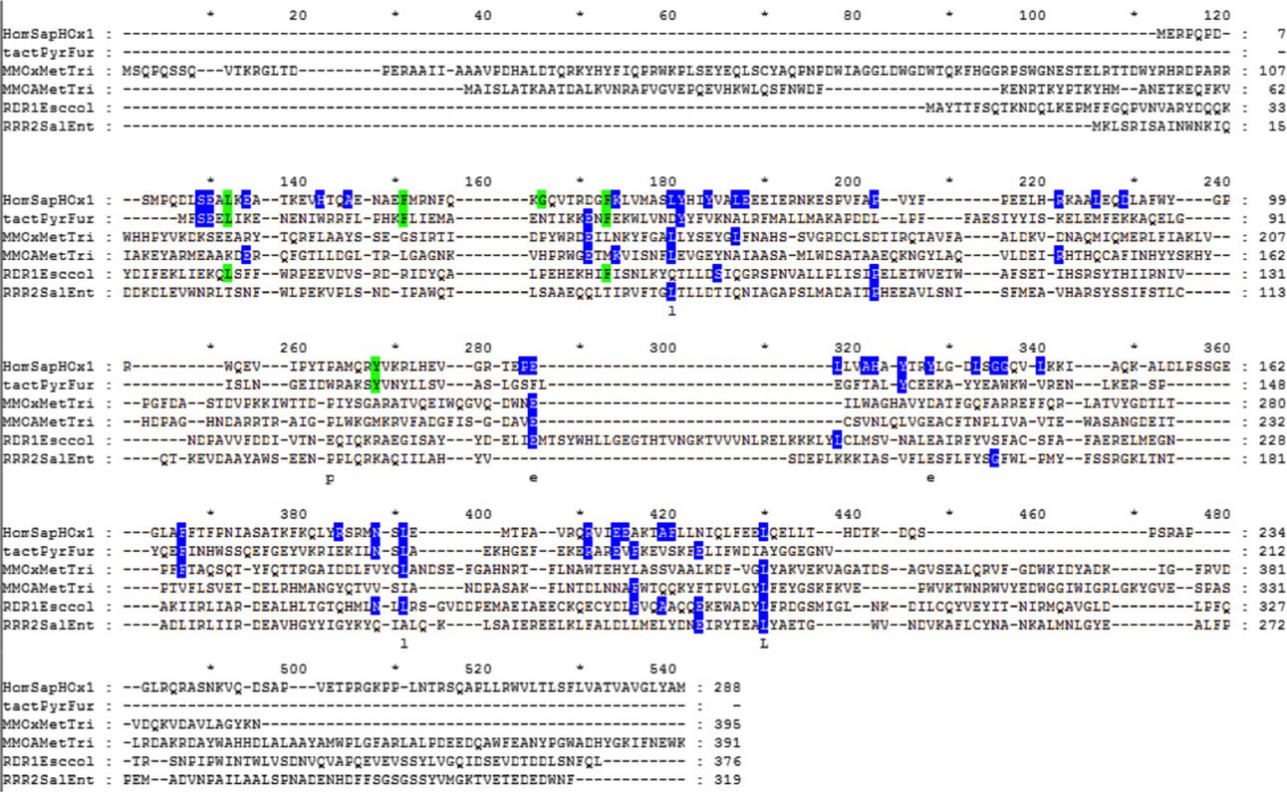
Summary alignment showing a representative sequence for each group of HO homologues: human HO1 (HomSapHOx1) as a HO,* Pyrococcus furiosus* thiaminase (tactPyrFur) as a thiaminase, the alpha (MMOAMetTri) and beta chains (MMOxMetTri) of the MMOH from *Methylosinus trichosporium* as MMOH chains, the RNR beta chain from *Escherichia coli* (RDR1Esccol) as a RNR1A, and the RNR R2F protein from *Salmonella typhimurium* (RRR2SalEnt) as a RNR1B. The entire alignment, which contains 472 protein sequences, is found in Fig. S1. Residue positions are colored based upon their conservation in the entire alignment (Fig. S1) as follows: green = 60–79% conserved and blue = 40–59% conserved. Indel (gap) positions from the entire alignment (Fig. S1) are retained to allow correlation with index position numbers (numbers shown above the alignment columns) that are noted within the text.

**Figure 2:**
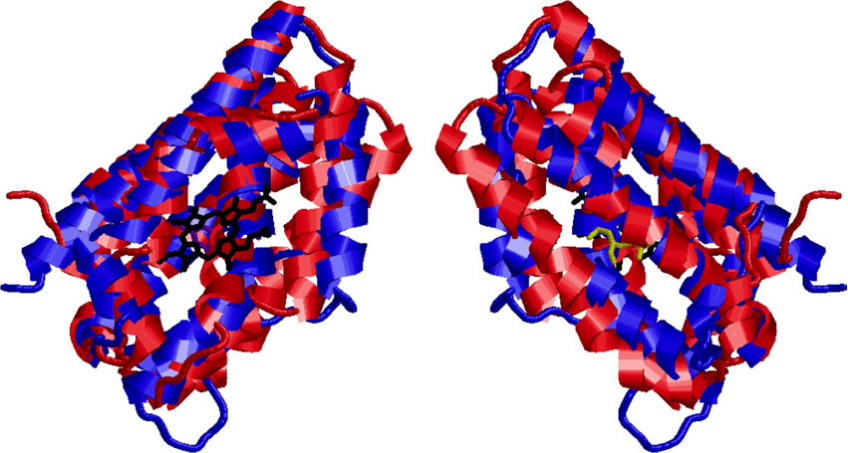
Structural alignment of human HO1 (blue, PDB ID: 1N45) and TenA thiaminase from *Bacillus subtilis* (red, PDB ID: 2QCX) performed using jFATCAT, shown in front and back view (180° rotation). The bound heme ring in human HO1 is shown in black, and the bound 4‐amino‐5‐hydroxymethyl‐2‐methylpyrimidine product in TenA is shown in yellow. Note how the bound ligands lie in pockets on opposite faces of the molecules.

A total of 472 sequences were aligned (Fig. 1) using tertiary structural alignment as a guide (entire alignment available in Fig. S1). Above each amino acid position column is an index number, which is numbered concurrently from the beginning of the alignment; these index numbers will be used to reference each position throughout this manuscript. Two hundred of the sequences analyzed were HOs from animal, plant, and bacterial species; 141 of the sequences were thiaminases; 41 of the sequences were MMOHs (30 alpha chains and 11 beta chains); and 90 of the sequences were Class I RNRs. MAPSCI structural alignment 24 indicates that the tertiary structures of all four homologues (human HO1, PDB ID: 1NI6; *P. furiosus* thiaminase, PDB ID: 1RTW; *Methylosinus trichosporium* MMOH alpha chain, PDB ID: 1MHY; and RNR beta chain from *E. coli*, PDB ID: 1XIK) are conserved with a core RMSD value of 1.37 Å and core size of 77, which represents the percentage of the length of the shortest protein aligned (1RTW at 201 amino acids) that have 100% conservation and are within 4.0 Å of each other in the final tertiary alignment. Interestingly, one can see that the active sites of HOs and thiaminases utilize different spatial coordination of the hydrophobic pocket, perhaps due to the differences in their enzymatic function (Fig. 2).

**Figure 3:**
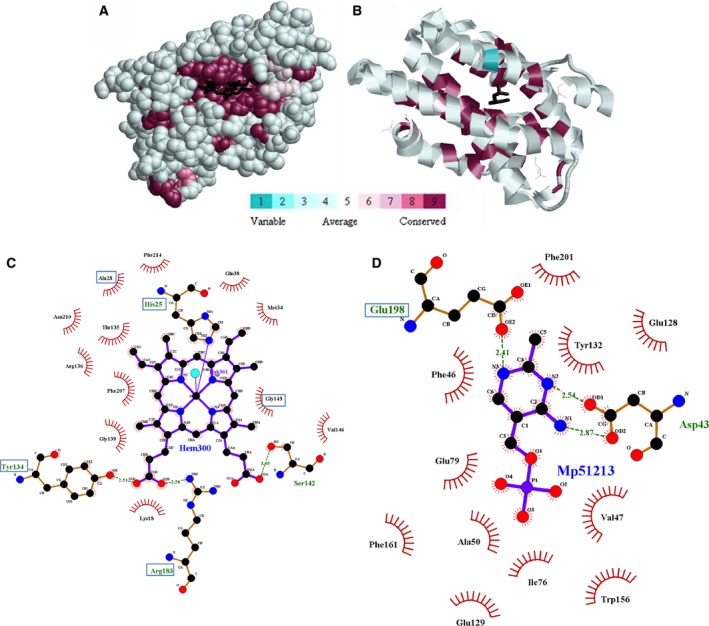
Evolutionarily conserved residue positions as determined by the CONSURF program 25. Shown are human HO1 (PDB ID: 1N45) with bound heme ring in black (A) and TenA thiaminase from *Pyrococcus furiosus* (PDB ID: 1RTW) with bound HMP‐P in black (B). Residue conservation scale is from CONSURF website. Note how most conserved positions surround the active site and protein core. The thiaminase is shown as a ribbon diagram as the HMP‐P is more buried and would be harder to see. ligplot+ diagrams 26 are shown to highlight residues that contact heme (designated Hem300) in human HO1 (C) and HMP‐P (designated Mp51213) in *P. furiosus* thiaminase (D) (similar to figure previously published 17). Blue boxes surrounding the residue names indicate that these residues are at least 40% conserved in the entire amino acid sequence alignment. Most of the residues in each active site are not conserved in the entire alignment due to the diversity of the structural homologues aligned.

The overall residue conservation within the HO structural homologues was low. No residues showed conservation of 80% or higher in the entire alignment, five residues exhibited conservation of 60% or higher, and 40 additional residues displayed conservation of 40% or higher. The majority of these conservations were found within the α‐helices. ConSurf analysis 25 was used to visualize these evolutionary conservations. In human HO1 (hHO1, sequence HomSapHOx1, PDB ID: 1N45) and the thiaminase from *P. furiosus* (PfThiaminase, sequence tactPyrFur, PDB ID: 1RTW), highly conserved residues were located in the core of the structure (Fig. 3). In hHO1, these residues conserved in the entire alignment line the heme‐binding site (Fig. 3A). Alignment index positions (Fig. 1) for each residue are provided in curved brackets. These conservations include His25{142} which coordinates the heme iron (Fig. 3C). Tyr134{325} and Arg183{384} form hydrogen bonds to the porphyrin ring. Mutations of the equivalent residue at index 384 in *Corynebacterium diphtherae* HO, Arg177{384}, caused a significant decrease in catalytic rate 27. Ala28{145} and Gly143{335} make hydrophobic contacts to the porphyrin ring. A G143H mutation inactivated both mouse and human HO1 28, 29. Tyr58{184} is part of a larger hydrogen bonding network, which will be discussed below 30. Note that the degree of conservation in the entire alignment, highlighted in Fig. 1, is influenced by the number of sequences for each homologue. For example, HOs represent 200 of the total 472 amino acid sequences aligned. In PfThiaminase, several conserved residues also lined the binding site for HMP‐P (Fig. 3B). These residues include Phe22{151} which packs with Phe36{163} near the end of the active site pocket. The hydroxyl of Tyr105{268}, which is near the back of the active site pocket, hydrogen bonds with the side chain carboxylate of Glu198{423}, which coordinates the bound HMP‐P (Fig. 3D) 17. Y112F and E205A mutations at indices 268 and 423, respectively, in *B. subtilis* TenA (BsThiaminase) both caused significant decreases in catalytic function 21. None of the other residues that contact HMP‐P are conserved above 60% in the entire alignment, perhaps because thiaminases represent only a quarter of all aligned sequences.

Five residue positions are conserved above 60% in our entire alignment: Leu17{132}, Phe33{151}, Gly40{166}, Phe47{173}, and Tyr114{268} (hHO1 residue identity with alignment index position in curved brackets). Four of five residues are involved in hydrophobic packing, as observed in the hHO1 structure 30. Leu17{132} is 69% conserved overall and was leucine in thiaminases, RNR1As, and HOs except plant species. This position also has 84% similarity with aliphatic hydrophobic residues (I, V, L, and M) throughout the entire alignment. This residue is found in α‐1 and is involved with hydrophobic packing with Ala203{416} (not conserved) and Tyr134{325} (44% conserved), which is located in the heme‐binding pocket of HO. Phe33{151} is 63% conserved overall and is found in thiaminases and HOs except plant species. This residue is found in the proximal helix of HOs and functions in hydrophobic packing with Val50{176} (not conserved). Gly40{166} is 63% conserved overall and is found in thiaminases and HOs except plant species. This residue is found in the turn between α‐1 and α‐2 30. Conserved glycines are often found in turns in enzyme structures or at positions where a side chain would lead to steric conflicts in many enzyme families 31, 32, 33, 34. Phe47{173} is 71% conserved overall and is found in RNR1As, most thiaminases, and HOs except plant species. This residue is found in the beginning of α‐2 and is involved in hydrophobic packing with Ala151{348} (not conserved) that is found in the distal helix of HOs. Tyr114{268} is 66% conserved overall and is found in all HOs and most thiaminases except proteins identified as Tregs. This residue is found in α‐4 in hHO1 30. In hHO1, the side chain hydroxyl of Tyr114{268} is 3.1 Å from NH1 of Arg136{327} (not conserved) from the distal helix of HOs, and 3.0 Å from ND1 of Asn210{423}. In PfThiaminase, the side chain hydroxyl of Tyr105{268}, which is part of the catalytic triad 21, is 2.7 Å from the side chain carboxylate of Glu198{423}, which contacts the bound HMP‐P 17. Glutamate is 46% conserved at index 423 in the alignment, primarily in all RNRs and thiaminases except Tregs.

Fourteen additional residue positions also display amino acid similarity of 60% or higher within these structural homologues. All but one of these positions are hydrophobic residues involved in hydrophobic packing. Index position 152 (Met34 in hHO1) exhibits 79% similarity of aliphatic hydrophobic residues (I, V, L, and M) in HOs except plant species, thiaminases except Tregs, and RNR1As. Met34{152} faces α‐meso‐face of heme in the hHO1 structure 30. Index 168 (Val42 in hHO1) shows 62% similarity of aliphatic residues in HOs except plants species and most thiaminases. Index 177 (Met51 in hHO1) displays 63% similarity of aliphatic residues in a few HOs, all thiaminases, and RNR1As. Index 230 (Leu93 in hHO1) shows 71% similarity of aliphatic residues in all HOs, a few thiaminases, MMOHs, and all RNRs. Index 272 (Leu118 in hHO1) exhibits 93% similarity of aliphatic residues in all HOs, most thiaminases, and all RNRs. Index 320 (Val130 in hHO1) shows 84% similarity of aliphatic residues in most HOs, most thiaminases, the beta chain of MMOHs, and RNR1As. Index 340 (Leu147 in hHO1) shows 68% of aliphatic residues in all HOs, some thiaminases, and the alpha chain of MMOHs. This residue is located on the distal helix and contacts with heme in HOs 30. The CD2 atom of Leu147{340} forms a hydrogen bond with the NE atom of Gln38{156} 35. Val139{340} in PfThiaminase is about 3.6 Å from Leu23{152}. Index 367 (Phe166 in hHO1) displays 71% similarity of aromatic residues with mostly phenylalanines in HOs and tryptophans in thiaminases. Index 387 (Met186 in hHO1) exhibits 85% similarity of aliphatic residues in HOs, MMOHs, RNR1As, and some thiaminases. Met186{387} is located about 6.6 Å from the heme molecule in hHO1. In PfThiaminase, Leu172{387} is located 4.2 Å from Phe122{320}.

Index 388 (Asn187 in hHO1) shows 82% similarity of aspartate or asparagine (asparagine is 58% conserved in the entire alignment) in HOs, RNR1As, and some thiaminases. Index 388 appears to maintain protein folding. The side chain OD1 atom of Asn187{388} is 3.2 Å away from the main chain nitrogen of Leu13{128} in hHO1. In PfThiaminase, the ND atom of Asn173{388} is 3.0 Å from the hydroxyl of Ser3{129}, and the OD1 atom is 2.8 Å from the main chain nitrogen of Ser3{129}. Index 391 (Leu189 in hHO1) displays 85% similarity of aliphatic residues in HOs, MMOHs, RNR1As, and some thiaminases. Index 412 (Val199 in hHO1) exhibits 61% similarity of aliphatic residues in most HOs, some thiaminases, and the alpha chain of MMOHs. Index 427 (Phe214 in hHO1) shows 63% similarity of aromatic residues (phenylalanine or tryptophan) in HOs except plant species and most thiaminases. It is found on the wall opposite the α‐meso‐face of heme in hHO1 35. Index 430 (Leu217 in hHO1) displays 74% similarity of aliphatic residues in HOs, MMOHs, RNRs, and a few thiaminases.

### Conserved sequence motifs

**Figure 4:**
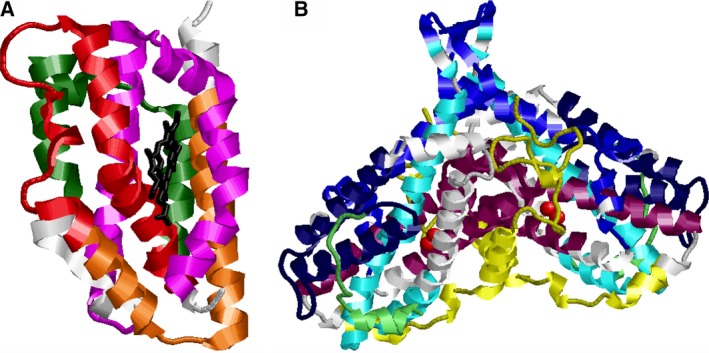
Conserved motifs found in the monomer of human HO1 (A) and a dimer of R2 protein of *Escherichia coli* RNR (B). Motifs 1 (red), 2 (pink), 3 (orange), and 6 (dark green) comprise the active site of HO (A). Motifs 5 (purple) and 9 (cyan) line the binding site with the diiron cluster (red) (B). Motifs 4 (yellow), 5, 9, and 10 (blue) form intersubunit contacts within the RNR dimer. Motifs 7 (light green) and 8 (navy blue) are found at the surface.

Ten conserved sequence motifs were identified using the meme program 36) (Table 1). Motifs 1, 2, 3, and 6 are only found in HOs. All four motifs line the heme‐binding pocket (Fig. 4A). Motif 1 is comprised of the distal helix, which includes the heme iron ligand His25{142}, and motif 2 constitutes the proximal helix of HOs 30. Motifs 4, 5, 7, 8, 9, and 10 are found only in the RNR1A enzymes. In EcRNR1A, motifs 4, 5, 9 and 10 from intersubunit contacts within the dimer, while motifs 5, which includes the iron ligands Asp84{184}, Glu115{219}, and His118{223}, and 9, which includes the iron ligand Glu204{328}, line the binding site with the diiron cluster 19 (Fig. 4B). None of the top ten motifs were found in thiaminases, MMOHs, or RNR1Bs.

**Table 1:** Ten most conserved sequence motifs in the HO homologues

Motif number	Motif regular expression	Indices	Group
1	EPELLVAHAxYTRY[LM]GDLSGxGQVLKK[VI]xxAQ[RK]ALKLPS[TS]GExxxGL[QA]F[YF]	283–368	HO
2	TKE[VAS]H[DE][RQ]AExNTxxx[QE]F[VM]K[DN]F[LQ]KG[QRN][IV][KT][KR]xE[LG]FKL[AV][TM][AVT]x[AS]LY[FH][TI]YSALEEE	138–190	HO
3	[DEP][NG][IV][SD][NS]A[QT][QK]FKQLYR[AS]R[ML]NxAL[ED]xxxxxxxxx[LM][DT]PExx[TE]KER[IV][VL]EEA[NK]KAF	371–420	HO
4	KNDQLKEPMF[FL]GQ[PS]VNVARYDQQK[YFH][ED][IV]FE[KR]LIEKQLSFFxxWRPEEVDVS	97–146	RNR1A
5	SNLKYQTLLDSIQxGRSPNVALLPL[VI]S[IL]PExxLETW[IV]ETxxW[AS]FSETIHS	175–224	RNR1A
6	ALE[KQ]D[LM][EA][YF][FW]YxxxxxG[EP][DN]xxxxW[REKQ]E[QK]xx[IV][PQ][CY][ST][EP]AxxxxxT[QR][KR]YV[DE]R[IL]H[EY]V	225–275	HO
7	G[AT][RKT][QS]NPIPWIN[TA]WL[SV]SDNVQVAPQE[VA]E[VI]SSYLVGQID[SNA]EV[DNS][AT]DD[LF][GS]DF[EQ]L	481–532	RNR1A
8	AxAEQEKEWxA[ED]YLF[KRQ]DGSMIGLxxNKxxDILCQYVEYITxN[IL]RMQAVGL	419–466	RNR1A
9	LRELKKxKLYLCLMSVxxNALEAIRFYVSFACxSFAxxFAERELMxxEGN	309–354	RNR1A
10	DP[SA][VIQ]VFDDIV[TE]NE[QH]I[LQI]KRA[EG][DGS]I[SA][HC]YYDDLIE[AM]T[SNAQ][YLD][YW][HN][LR][LYH]GEGTHT[VI]NG[KE][TE]	249–304	RNR1A

mast was used to search for matches in the protein database using the motifs. The strongest matches were RNRs (lowest *E*‐score = 3.4e‐154), followed by HOs (lowest *E*‐score = 7.5e‐30). As noted, the ten motifs identified in this study were only present in RNRs and HOs. Two weaker scoring matches that occurred multiple times were the prokaryotic MreB rod‐shape determining bacterial actin protein (A0A1H1VW64_9PSED) and the SecB protein‐export protein (A0A062MXT3_9GAMM). MreB showed homology to motifs 3 and 10; however, the *E*‐score was only 0.068. The MreB protein from *Caulobacter crescentus* (PDB ID: 4CZM) 37 did not show any structural homology to HOs or its related homologues. SecB showed homology to motifs 6 and 10; however, the *E*‐score was only 1.7. The SecB chaperone protein from *E. coli* (PDB ID: 1QYN) 38 also did not show any structural homology to HOs or its related homologues.

### Phylogenetic analysis

**Figure 5:**
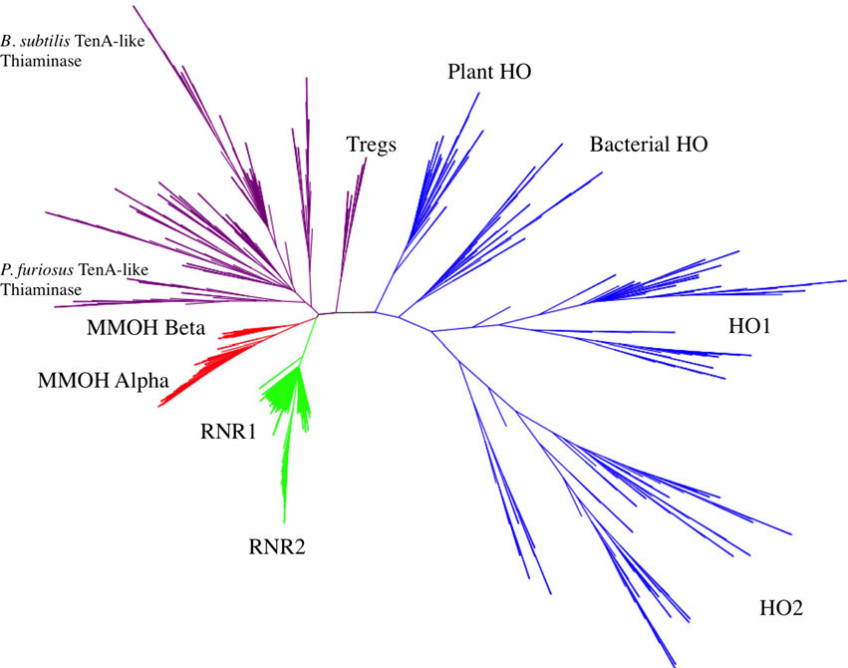
Unrooted phylogenetic tree of the HO homologues. Branches are color‐coded based on enzyme type: blue = HOs; purple = thiaminases; red = MMOHs; and green = RNRs. Specific subgroups within each enzyme group are labeled.

An unrooted neighbor‐joining phylogenetic tree of the HO structural homologues (Fig. 5) was generated from the entire amino acid sequence alignment. This neighbor‐joining method was chosen because it has been shown to yield excellent evolutionary relationships in some families 34, 39. Maximum‐likelihood and parsimony methods require excessive computations for large data sets. A bootstrapped parsimony tree using only 50 data sets (Fig. S2) had similar sequence groupings and group arrangements to the neighbor‐joining tree using 250 replicates. The neighbor‐joining tree was subsequently used as the basis for assigning each sequence into a group for group entropy analysis. HOs, thiaminases, MMOHs, and RNRs cluster into distinct clades and these four groups were used for group entropy analysis. Groups were named based upon the representative tertiary structures present in each clade. MMOHs and RNRs are more closely related to each other, but both are more distantly related to HOs.

Distinct subgroups were also evident for each group. For example, in HOs vertebrate HO1s and HO2s, as well as plant and bacterial HOs, were isolated in distinct clades. In thiaminases, three distinct subgroups were apparent. There was a group of sequences similar to PfThiaminase and another group similar to BsThiaminase, as previously observed 23. There was also a subgroup of Tregs identified in the thiaminase group. It is possible that these Tregs may not possess the enzymatic function of other thiaminases as they are missing certain residues found in other thiaminases, such as Glu205{418} and Cys135{322} in BsThiaminase 21, where Tregs have threonine and alanine in those positions, respectively. In MMOHs, the alpha and beta chains cluster separately. In the RNRs, both RNR1As and RNR1Bs are located in distinct clades.

### Group entropy analysis of HOs

The GEnt program 40 was used to calculate ‘Group Entropy’ and ‘Family Entropy’ values for each position in the alignment. Residue positions studied here had the highest Group Entropy scores within each homologue group, indicating they are well conserved within its group, and low Family Entropy scores, indicating they are not well conserved throughout the entire alignment. These residues would indicate unique residue conservations within the enzyme group. For the current study, residue positions examined had a Group Entropy score of at least 10.0, with Family Entropy score of no more than 3.0. The GEnt program utilizes the Kullback–Leibler method to calculate a divergence measure to detect covariance. It was chosen as it allows the user to define each group, to which the user can assign sequences from an alignment. Also, GEnt does not consider positions with gaps 40. The GEnt program has been used to identify group‐specific conservations in Class 3 aldehyde dehydrogenases 40 and NDP‐sugar dehydrogenases 34.

Eight residues were found to have the highest Group Entropy scores in the HO group (Table 2, complete GEnt results can be found in Table S1). Examination of residues was made with hHO1 (sequence HomSapHOx1, PDB ID: 1N45). The residue position with the highest Group Entropy score is index 142. His25{142}, which is fully conserved on the proximal helix in all HOs, is the critical heme iron ligand 30 (Fig. 3C). A H25A mutation in hHO1 led to inactivation of the enzyme 41. Next, Tyr58{184} interacts with several polar residues near pyrrole B 30. In hHO1, the hydroxyl of Tyr58{184} hydrogen bonds with the side chain carboxylate of Asp140{332} and is 3.5 Å from the NE atom of Arg136{327}. Index 322 had the third highest Group Entropy score. The NE2 position of His132{322}, which is invariant in HOs, forms a salt bridge to the side chain carboxylate of Glu202{415} 30. Next, Arg136{327} is a residue that is invariant in the HOs of vertebrates but is isoleucine for plant species. The side chain of Arg136{327} is part of a hydrogen bonding network that also includes Asp140{332}, Asn210{423}, Tyr58{184} (also identified by GEnt above), and Tyr114{268}, which is 66% conserved in the entire alignment. This network may serve to help activate the oxygen in the catalytic mechanism 35. A D140A mutation in hHO1 also converts the enzyme into a peroxidase, illustrating the critical nature of this hydrogen bond network 42.

**Table 2:** Group entropy analysis of HOs

Index	Residue identity^a^	Group entropy	Family entropy	Highest group residue	Common thiaminase residue	Common MMOH residue	Common RNR residue
142	His25	17.164	2.800	His	Trp	Leu	Glu, Lys
184	Tyr58	16.526	1.812	Tyr	Leu, Val	Glu	Asp
322	His132	15.654	2.012	His	Ala	Asn	Ser
327	Arg136	13.071	1.497	Arg	Cys	Gly	Leu
188	Glu62	12.683	1.065	Glu	Ala	Phe, Ile	Gly, Asn
383	Tyr182	12.348	1.449	Tyr	Leu, Gln	Gly	Thr, Ile
325	Tyr134	12.334	1.924	Tyr	Leu	Leu	Asn, Val
371	Pro170	12.150	1.094	Asp	Tyr, Trp	Ile, Val	Ile

^a^ Residue identity in HomSapHOx1.

Glu62{188} is conserved in all but one HO sequence, which has a conservative replacement with an aspartate. The side chain carboxylate of Glu62{188} is located 3.4 Å from the main chain nitrogen of the conserved Arg85{222}, acting to maintain enzyme structure. Next, Tyr182{383} is conserved in the HOs of vertebrates but is valine in plants. Its side chain hydroxyl is nearly 4 Å from the main chain nitrogen of Tyr134{325} and the main chain oxygens of Ala133{323} and Val77{205}. Tyr134{325}, which is conserved in all HOs, is located on the distal helix and contacts the beta edge of heme 30, 35. Lastly, Pro170{371} lies on a surface loop and is a hydrophobic residue in the other groups.

### Group entropy analysis of thiaminases

Eight residue positions in thiaminases had the highest Group Entropy scores (Table 3, complete GEnt results can be found in Table S2). Residues noted here were from PfThiaminase (sequence tactPyrFur, PDB ID: 1RTW). Index 142 had the highest Group Entropy score in thiaminases. Trp14{142} is involved in hydrophobic packing and is located where the heme‐binding site is in HOs. The NE1 position of Trp14{142} also hydrogen bonds to the side chain hydroxyl of Ser195{420}. Next, Val47{184} forms the wall of the HMP‐P binding pocket 17 (Fig. 3D). The side chain carboxylate of Asp43{180} forms hydrogen bonds with the amino groups of the HMP‐P ligand. A D44A mutation at index 180 in BsThiaminase caused a significant decrease in catalytic function 21. Index 371 had the fourth highest Group Entropy score. Trp156{371} also lines the HMP‐P binding pocket, making hydrophobic contact with the ligand. Next, Phe46{183} is at an index position of aromatic residues in thiaminases. The pyrimidine ring of HMP‐P is located between the side chains of Tyr132{332} and Phe46{183} 17. Interestingly, a Y47F mutation at index 183 in BsThiaminase leads to a significant impairment of catalytic function 21. Also, a F47Y mutation at index 183 in *Helicobacter pylori* TenA disrupts the helical folding of helices D and E 43.

**Table 3:** Group entropy analysis of thiaminases

Index	Residue identity^a^	Group entropy	Family entropy	Highest group residue	Common HO residue	Common MMOH residue	Common RNR residue
142	Trp14	17.993	2.800	Trp	His	Leu	Glu, Lys
184	Val47	14.133	1.812	Leu	Tyr	Glu	Asp
180	Asp43	12.746	1.399	Asp	Leu, Ser	Leu	Gln, Leu
371	Trp156	12.734	1.094	Trp	Asp	Ile, Val	Ile
183	Phe46	11.989	1.146	Tyr	Ile, Val	Gly	Leu
430	Ala205	11.192	1.693	Ala	Leu, Ile	Leu, Ile, Met	Leu
367	Phe152	11.161	2.534	Trp	Phe	Val	Ile, Leu
149	His20	10.376	1.339	His	Ala, Thr	Arg	Asp

^a^ Residue identity in tactPyrFur.

Ala205{430} is near the C terminus on the surface of the protein, but not at the subunit interface. Index 430 is a position that is an aliphatic residue in 74% of all aligned sequences. Next, Phe152{367} lies at a position in thiaminases that is predominantly aromatic. Its side chain is nearly 8.6 Å from the bound HMP‐P. Lastly, His20{149}, which is 90% conserved in the aligned thiaminases, forms a salt bridge with the side chain carboxylate of Asp203{428} 17.

### Group entropy analysis of MMOHs

GEnt analysis of MMOHs revealed that ten residues have the highest Group Entropy scores (Table 4, complete GEnt results can be found in Table S3). Residues from both the alpha (sequence MMOAMetTri) and beta chains (sequence MMOxMetTri) were researched in the MMOH from *M. trichosporium* (PDB ID: 1MHY). Several residues identified here make intersubunit contact. The highest scoring index position is index 265. Trp181{265} from the alpha chain makes hydrophobic contact with Pro61{74} from the beta chain. The residue from the beta chain at index 265 is Tyr227{265}, which is involved in hydrophobic packing within the beta chain. Next, Leu82{142} is conserved in all MMOHs except for two that have alanine. This index position is involved in hydrophobic packing for both alpha and beta chains. The third highest Group Entropy position is Met184{268} and it lines the hydrophobic cavity where the diiron cluster is located in the alpha chain 16. Index 268 is a tyrosine in 66% of all sequences in the entire alignment. Glu114{184}, which is fully conserved in MMOHs, coordinates the FE1 iron atom and is one of four residues that coordinate the iron and hydroxo ligands 16. The fifth highest Group Entropy score is Tyr115{185}, which is invariant in MMOHs. The hydroxyl of Tyr115{185} in the alpha chain is located 3.4 Å from the main chain nitrogen of Asp176{202} in the beta chain, making intersubunit contact. Tyr160{180} in the beta chain is about 5 Å from Trp37{74} and Phe35{72} in the alpha chain.

**Table 4:** Group entropy analysis of MMOHs

Index	Residue identity^a^	Group entropy	Family entropy	Highest group residue	Common HO residue	Common thiaminase residue	Common RNR residue
265	Trp181	15.658	1.167	Trp	Met, Thr	Ala, Asn	Ile, Leu
142	Leu82	14.411	2.800	Leu	His	Trp	Glu, Lys
268	Met184	14.148	2.802	Met	Tyr	Tyr, Phe	Arg, Lys
184	Glu114	13.946	1.812	Glu	Tyr	Val, Leu	Asp
185	Tyr115	13.513	0.836	Tyr	Val, Ser	Lys, Asp	Ser, Thr
229	Phe153	11.619	1.376	Phe	Asp	Glu	His, Ser
365	Pro233	11.396	0.621	Pro	Leu, Ile	Gln	Ala
168	Arg98	11.257	1.097	Trp	Val, Ile	Leu	His, Ala
169	Trp99	11.067	0.887	Trp	Thr, Ser	Glu, Asp	Glu
183	Gly113	10.025	1.146	Gly	Ile, Thr	Tyr	Leu

^a^ Residue identity in MMOAMetTri.

Next, Phe153{229} in the alpha chain is involved in hydrophobic packing with Leu55{68} from the beta chain, making intersubunit contact. The seventh highest scoring index position is Pro233{365}, which is fully conserved in both alpha and beta chains of MMOHs. This residue is at the beginning of α‐F that lines the diiron cluster‐binding site, likely positioning the helix. Next, the side chain of Arg98{168} in the alpha chain functions by forming a salt bridge to Asp365{515}. In the beta chain, Trp143{168} is involved in hydrophobic packing. The ninth highest scoring index position is index 169. Trp99{169} is a residue that is involved in hydrophobic packing in the alpha chain. The last residue examined is Gly113{183}. This residue is fully conserved in the alpha chain and lines the pocket for the diiron cluster. The lack of a side chain on this residue could be important for positioning of the iron atoms.

### Group entropy analysis of RNRs

Eleven index positions displayed the highest Group Entropy scores in RNRs (Table 5, complete GEnt results can be found in Table S4). Residues were examined using EcRNR1A (sequence RDR1Esccol, PDB ID: 1XIK). Trp49{138} had the highest Group Entropy score and is invariant among all RNRs. Trp49{138} likely serves as the tryptophan radical, providing an electron to completely reduce the dioxygen. It also hydrogen bonds to the iron ligand His118{223} via Asp237{375} 19. Arg149{268} is the next residue and is also fully conserved in RNR1As but is lysine in RNR1Bs. The NE position of Arg149{268} is located 3.3 Å from the side chain carboxylate of Glu283{423}. Arg149{268} also stacks against Trp286{426} 19, which is also identified by GEnt. Trp286{426} is invariant in RNR1As but is tyrosine in RNR1Bs. The NE1 position of Trp286{426} hydrogen bonds with the hydroxyl of Ser75{175}. Trp286{426} is also involved in hydrogen bond changes in a S211A mutant 19.

**Table 5:** Group entropy analysis of Class I RNRs

Index	Residue identity^a^	Group entropy	Family entropy	Highest group residue	Common HO residue	Common thiaminase residue	Common MMOH residue
138	Trp48	20.487	1.532	Trp	Thr	Val, Ala	Gln
268	Arg149	18.774	2.802	Arg	Tyr	Tyr	Met, Ala
336	Phe212	17.436	1.899	Phe	Gly	Gly	Ile
426	Trp286	17.000	1.784	Trp	Ile, Val, Leu	Phe	Phe, Ala, Val
184	Asp84	16.747	1.812	Asp	Tyr	Leu	Glu
142	Glu52	15.901	2.800	Glu	His, Ala	Trp	Leu
135	Phe47	15.211	0.834	Phe	Ala, Gly	Thr, His, Arg	Arg
187	Gln87	14.911	1.548	Gln	Leu	Phe	Ala
179	Tyr79	14.360	1.417	Tyr	Ser, Ala	Gln	Ala, Phe
181	Thr81	14.134	1.563	Thr	Tyr	Tyr, Ser	Glu
327	Leu203	13.930	1.497	Leu	Arg	Cys	Gly

^a^ Residue identity in RDR1Esccol.

Phe212{336}, which is fully conserved in all RNRs, forms a conserved hydrophobic pocket with residues Phe208{332} and Ile234{371} for the binding of dioxygen 19. Next, Asp84{184} is invariant in all RNRs and coordinates one of the iron ligands 19. The position with the sixth highest Group Entropy score is Glu52{142}, which is on the surface of the molecule near the subunit interface. Analysis of an E52Q mutant in the beta chain of RNR1A appears to suggest that Glu52{142} is critical for the conformational change at the α/β subunit interface that initiates radical formation 44. Next, Phe47{135}, which is fully conserved in all RNRs, ring stacks with Trp286{426} between subunits. Another residue identified by GEnt that is invariant in all RNRs is Gln87{187}. The OE1 position of Gln87{187} is located 2.9 Å from the side chain carboxylate of Glu204{328}, which is an iron ligand 19. Next, the hydroxyl of Tyr79{179} hydrogen bonds to the side chain carboxylate Glu283{423}, which also interacts with Arg149{268} noted above. The Cα of Thr81{181}, which is fully conserved in all RNRs, is 3.8 Å away from the side chain of Tyr122{227}, which is suggested to be the radical in the enzyme mechanism 19. Lastly, Leu203{327} is involved in hydrophobic packing with the side chain of Gln87{187}, also identified by GEnt.

### Alternate methods for determining functional residues

The evolutionary trace method was developed to identify critical residues in active sites and clusters of residues at functional interfaces in proteins. The program generates a dendrogram of aligned sequences and creates evolutionary partitions that divide the tree into distinct families and subgroups. Residues unique to each group, both buried and on the surface, can be identified 45, 46. For HOs, partition 2 groups all HOs in one group, but does not identify any group‐specific residues. It is not until partition 8, where HOs are divided into 16 subgroups, that six group‐specific residues are identified (Table 6). Of these, only His25{142} and Tyr58{184} are among the residues in HOs with the highest Group Entropy scores (above). After the tenth and final partition, all but one, Pro170{371}, of the highest scoring residues in GEnt are identified by evolutionary trace, but there are an additional 28 residues also identified in the 21 HO subgroups. This highlights one of the advantages of GEnt that one can define each group with representative sequences, instead of following arbitrary partitions.

**Table 6:** Index positions identified by functional residue identification programs in HOs

GEnt	Evolutionary trace	Jensen–Shannon divergence	Property entropy	VN entropy	Relative entropy	Shannon entropy	Sum of pairs
142	142	142	184	173	142	142	142
184	173	184	187	184	321	184	184
188	179	229	188	188	322	187	187
322	184	321	229	198	323	188	198
325	328	322	230	229	325	222	222
327	332	323	319	232	327	226	229
371		325	320	268	328	229	268
383		327	321	284	332	268	322
		328	322	322	334	321	325
		329	325	325	335	322	328
		330	326	328	336	325	335
		332	328	330	337	326	336
		333	335	332	367	327	337
		334	336	334	381	328	367
		335	337	335	384	330	368
		336	338	336		332	370
		337	340	337		333	384
		367	348	367		334	415
		384	367	368		335	420
			368	370		336	
			370	381		337	
			384	384		367	
			387	388		381	
			413	415		384	
			415	420		415	

Evolutionary trace also does not result in any group‐specific residues from partition 2 of thiaminases, where thiaminases were divided into only two groups. It is not until partition 8, where thiaminases are divided into 20 subgroups, that six group‐specific residues are identified (Table 7). Of these, only Trp14{142} and Val47{184} are among the residues in thiaminases with the highest Group Entropy scores (above). It is also interesting to note that the residue positions identified by evolutionary trace in HOs are the same ones identified in thiaminases. This coincides with the fact that HOs and thiaminases are on neighboring clades in the phylogenetic tree (Fig. 5).

**Table 7:** Index positions identified by functional residue identification programs in thiaminases

GEnt	Evolutionary trace	Jensen–Shannon divergence	Property entropy	VN entropy	Relative entropy	Shannon entropy	Sum of pairs
142	142	142	142	142	142	142	142
149	173	149	149	149	149	149	149
180	179	151	151	150	151	151	151
183	184	152	152	151	152	152	152
184	328	166	155	152	166	166	166
367	332	176	166	166	179	168	173
371		179	168	173	180	173	176
430		180	173	176	326	176	177
		181	176	177	327	177	179
		183	177	179	329	179	180
		268	178	180	332	180	181
		326	179	181	364	181	183
		327	180	183	367	183	268
		332	181	268	426	184	327
		367	183	271	427	267	332
		423	184	326	428	268	364
		426	268	327	429	326	367
		427	271	332		327	423
		428	272	364		332	426
			323	367		364	427
			327	376		367	
			423	423		423	
			426	426		426	
			427	427		427	
			428	428		428	

Evolutionary trace partition 2 places MMOH alpha and beta chains in separate groups. Evolutionary trace partition 4, the last partition to have the alpha and beta chains in one group each, identified five group‐specific positions in the beta chain: Trp221{258}, Trp238{276}, Phe259{331}, Phe267{339}, and Asp277{351}. However, Trp221{258}, Trp238{276}, and Phe267{339} are at alignment positions that are primarily indels in other families. In the *M. trichosporium,* MMOH alpha chain partition 4 identified 18 positions (Table 8). However, 15 of these residues are at or above alignment index 468, which constitute the C terminus, and residues that extend beyond the length of all of the other homologues (beyond index 540 of the summary alignment in Fig. 1). Thus, similar to the residues in the beta chain at indel positions, most of these positions do not have residues in other homologues to which to compare. Of the residues identified in both chains by evolutionary trace, only Leu82{142} and Glu114{184} identified in the alpha chain score highly for Group Entropy in GEnt. Another feature of GEnt analysis is that it does not consider positions with gaps. Thus, most positions identified by evolutionary trace for MMOHs were not identified by GEnt.

**Table 8:** Index positions identified by functional residue identification programs in MMOHs

GEnt	Evolutionary trace^a^	Jensen–Shannon divergence	Property entropy	VN entropy	Relative entropy	Shannon entropy	Sum of pairs
142	142	184	173	125	168	171	184
168	173	185	184	171	169	184	185
169	179	222	185	184	224	185	218
183	184	224	187	185	263	186	263
184	211	263	206	186	265	187	282
185	236	265	218	206	351	218	350
229	247	282	220	218	354	219	351
265	276	326	255	255	365	222	353
268	277	328	264	263	454	224	354
365	324	329	265	265	455	263	365
	328	331	267	282	460	282	457
	331	332	282	285		285	459
	332	350	285	328		326	460
	346	351	319	329		328	504
	351	354	325	330		329	521
	458	365	326	331		331	526
	460		328	332		332	527
	461		330	333		334	529
			331	350		349	603
			350	351		350	
			351	353		351	
			353	354		353	
			354	365		354	
			365	409		365	
			431	431		411	

^a^ 1MHY alpha chain.

Evolutionary trace partition 2 places all RNRs in the same group, while partition 8 is the last to group RNR1A and RNR1B in one group each. Partition 2 did not identify any group‐specific residues in all RNRs. Partition 8 identified 12 group‐specific residues in RNR1As (Table 9). Partition 8 identified 11 group‐specific residues in RNR1Bs, all of which were also identified in RNR1As (Table 9). Of the residue positions identified in both RNR groups, indices 142, 179, and 184 were highly scoring for Group Entropy for all RNRs in GEnt.

**Table 9:** Index positions identified by functional residue identification programs in RNRs

GEnt	Evolutionary trace RNR1A	Evolutionary trace RNR1B	Jensen–Shannon divergence	Property entropy	VN entropy	Relative entropy	Shannon entropy	Sum of pairs
135	142	142	135	138	133	135	133	135
138	173	173	138	140	135	138	135	138
142	179	179	140	143	138	140	138	140
179	184	184	181	182	140	187	140	141
181	211	211	187	183	141	223	141	184
184	296	317	217	184	184	225	181	202
187	317	328	223	185	185	336	182	223
268	328	331	225	186	187	345	183	225
327	331	332	226	222	202	378	184	227
336	332	346	332	223	217	426	186	333
426	346	351	336	225	225	490	187	336
	351		345	226	226	491	223	345
			373	227	227		225	373
			375	228	228		226	375
			376	365	333		227	376
			379	367	336		333	379
				368	338		336	490
				369	345		345	
				370	375		365	
				371	376		368	
				372	422		371	
				373	423		373	
				375	451		375	
				376	454		376	
				378	490		377	

In addition, six other algorithms were used to identify functional residues in HOs, thiaminases, MMOHs, and RNRs: Jensen–Shannon divergence, property entropy, VN entropy, relative entropy, Shannon entropy, and sum of pairs analysis 47. Each algorithm was used with combinations of all possible backgrounds and matrices. Only residues that were identified for all 21 combinations of backgrounds and matrices were reported as results for each method (Tables 6, 7, 8, 9).

### Common positions of group‐specific residues

Despite the fact that these homologues had different mechanisms, several common index positions were identified by various methods in multiple groups. These positions would represent critical sites of evolutionary differences between the homologues. Seven alignment index positions were identified in all four homologues: 142, 173, 179, 184, 268, 328, and 332. Index 142 had the highest Group Entropy score in GEnt in HOs and thiaminases, second highest in MMOHs, and sixth highest in RNRs. In HOs, His25{142} is the essential heme iron ligand 30. In thiaminases, Trp14{142} is involved in hydrophobic packing where the heme‐binding site is in HOs, but it also hydrogen bonds to the side chain hydroxyl of Ser195{420}. Leu82{142} in MMOHs is involved in hydrophobic packing for both alpha and beta chains. Thus, in thiaminases and MMOHs that lack a bound heme ring, this index position is involved in hydrophobic packing in the core of the protein. However, as indicated by evolutionary tracing, Glu52{142} in RNR1A and Lys35{142} in RNR1B are class‐specific, surface‐exposed residues. Both residues are near the subunit interfaces. Glu52{142} in the beta chain of RNR1A appears necessary for the conformational change at the α/β subunit interface that initiates radical formation 44.

The residues at index position 173 are involved in hydrophobic packing. Phe47{173} in hHO1 is at a position that is 71% conserved in the entire alignment and is a phenylalanine in RNR1As, most thiaminases, and HOs except plant species. MMOH alpha chains have Met103{173}. However, RNR1Bs have a polar Thr56{173}, which contacts Leu168{338}.

The residue at index 179 in hHO1, Ser53{179}, lies at the bottom of the heme‐binding pocket. In PfThiaminase, the side chain of Asn42{179} hydrogen bonds to the main chain nitrogen of Asp99{262}, maintaining protein structure. In the MMOH alpha chain, Phe109{179} packs with Trp181{265}. In EcRNR1A, the hydroxyl of Tyr79{179} hydrogen bonds to the side chain carboxylate of Glu283{423}. In *Sal. typhimurium* RNR1B, Gly62{179} lies in a helix preceding the iron ligand Asp67{184} 20.

Index 184 had the second highest Group Entropy score in HOs and thiaminases, fourth highest in MMOHs, and fifth highest in RNRs. In HOs, Tyr58{184} is involved in a hydrogen bonding network close to pyrrole B that may activate the oxygen in the catalytic mechanism 35. In thiaminases, Val47{184} forms the wall of the HMP‐P binding pocket 17. The residues in MMOH alpha chain, Glu114{184}, and RNR1A, Asp84{184}, both coordinate iron ligands 16, 19. Thus, this index position is critical for the unique function of each homologue.

Index 268 has the third highest Group Entropy score in MMOHs and second in RNRs. In the entire alignment, index 268 is a 66% conserved tyrosine. In MMOHs, Met184{268} lines the hydrophobic cavity where the diiron cluster is located in the alpha chain 16. In RNR1As, Arg149{268} forms a salt bridge with Glu283{423}, and also stacks against Trp286{426} 19, which is a residue position also identified by GEnt in RNRs. This might indicate a compensatory mutation in RNRs. Interestingly, although index position 268 did not score highly in group entropy in either HOs or thiaminases, several other functional residue identification methods identified index 268 in both homologues (Tables 6 and 7). This may have been due to the presence of a conserved tyrosine in both HOs and thiaminases. The side chain of Tyr114{268} in hHO1 also participates in the crucial hydrogen bond network 35. In PfThiaminase, the hydroxyl of Tyr105{268} hydrogen bonds with the side chain carboxylate of Glu198{423}, which coordinates the bound HMP‐P (Fig. 3D) 17. A Y112F mutation at index 268 in BsThiaminase caused a significant impairment of catalytic function 21.

The residue at index 328 serves critical roles in the active sites of most homologues. In PfThiaminase, the side chain carboxylate of Glu129{328} hydrogen bonds to phosphate of HMP‐P 17. The glutamate residues at index 328 in the MMOH alpha chain (Glu209{328}) and both RNR1A (Glu204{328}) and RNR1B (Glu158{328}) all coordinate iron ligands 16, 19, 20. However, in hHO1, the side chain of Tyr137{328} hydrogen bonds to the side chain carboxylate of Glu62{188}, maintaining enzyme structure.

The residue at index 332 also lies in the active sites of the homologues. In hHO1, Asp140{332} is part of the critical hydrogen bonding network 35. In PfThiaminase, the pyrimidine ring of HMP‐P is packed between the side chains of Tyr132{332} and Phe46{183} 17. In the *M. trichosporium* MMOH alpha chain, Thr213{332} is within 7 Å of the two iron atoms 16. Phe208{332} in RNR1As and Phe162{332} in RNR1Bs form a conserved hydrophobic pocket between the diiron cluster and tyrosyl radical 19, 20.

Two more index positions that scored high in Group Entropy in multiple homologues are indices 183 and 327. Index 183 scored fifth highest for Group Entropy in thiaminases and tenth highest in MMOHs. The pyrimidine ring of HMP‐P is packed between the side chains of Phe46{183} and Tyr132{332} in PfThiaminase 17. Mutations at this index position in BsThiaminase and *H. pylori* TenA thiaminase decrease catalytic activity and disrupt helical folding 21, 43. In MMOHs, Gly113{183} in the alpha chain lines the pocket for the diiron cluster 16. Index 183 was identified in RNRs by two of the eight methods performed. The Cβ of Leu83{183} in RNR1A is nearly 6.5 Å from the diiron cluster 19. In hHO1, Ile57{183} is approximately 10 Å from the bound heme 35.

Index 327 is fourth highest in Group Entropy for HOs and eleventh in RNRs. In hHO1, the side chain of Arg136{327} is part of the same hydrogen bonding network as Tyr58{184}, Tyr114{268}, and Asp140{332} (all noted above) that may help to activate the oxygen in the catalytic mechanism 35, demonstrating the critical nature of this network. However in RNRs, Leu203{327} is involved in hydrophobic packing with the side chain of Gln87{187}, another residue identified by GEnt in RNRs, perhaps indicating a compensatory mutation. Although index 327 did not score highly for Group Entropy for thiaminases in GEnt, Cys135{327} in BsThiaminase serves as the catalytic nucleophile for the enzyme. A C135A mutation completely inactivates the enzyme 21.

## Conclusions

While sharing some structural homology, HOs, thiaminases, methane monooxygenase hydrolases, and Class I RNRs share little sequence homology, with only five residue positions conserved in at least 60% of all 472 aligned sequences and fourteen more with amino acid similarities above 60% conservation. The most well‐conserved sequence motifs were present in HOs and RNRs. Phylogenetic analysis revealed that the four homologues were distinct, and these four groups were used to perform group entropy analysis, as well as several other methods to predict functional residues. Despite the amino acid sequence and functional differences in each homologue, several common residue positions appeared critical in determining the unique function of each homologue. In HOs, the residue at index 142 coordinated the heme iron, while residues at indices 184, 268, 327, and 332 all participated in an essential hydrogen bonding network. Residues at indices 173 and 328 helped to maintain enzyme structure, and the residues at indices 179 and 183 line the heme‐binding pocket 35.

In thiaminases, the residues at indices 142 and 173 are involved in hydrophobic packing. Index 179 maintains enzyme structure through a hydrogen bond. The residue at index 184 lines HMP‐P binding site. The residues at indices 183, 268, 328, and 332 help to bind HMP‐P, while the residue at index 327 acts as the catalytic nucleophile 17, 21.

In MMOHs, the residues at indices 142, 173, and 179 contribute to hydrophobic packing. Residues at indices 184 and 328 coordinate the iron atoms, while residues at indices 183, 268, and 332 line the active site 16.

In Class I RNRs, the charged residue at index 142 is located on the surface of the enzyme at the subunit interface and may be important for a critical conformation change. The residues at indices 173 and 327 are involved in hydrophobic packing. The residues at indices 179 and 268 form bonds that maintain enzyme structure in RNR1As. The residues at indices 184 and 328 coordinate the diiron cluster, while the residues at indices 183 and 332 line the active site 19, 20. The common index positions identified here represent potential evolutionary evidence that may explain the diversity of these enzymes despite being structurally homologous. These group‐specific residues, as well as the conserved residues throughout all four homologues, could also serve as excellent targets for site‐directed mutagenesis by others.

## Materials and methods

The procedure used here was similar to that previously published 34. The project began by downloading the amino acid sequences and tertiary structures of human HO1 (PDB IDs: 1NI6 & 1N45), human HO2 (PDB ID: 2RGZ), TenA from *P. furiosus* (PDB ID: 1RTW), Treg from *Streptococcus pneumoniae* (PDB ID: 1UDD), TenA from *B. subtilis* (PDB ID: 2QCX), HO‐like domain from *Shewanella denitrificans* (PDB ID: 3DDE), THI20 from *Sac. cerevisiae* (PDB ID: 3RM5), MMOH from *M. trichosporium* (PDB ID: 1MHY), RNR R2F from *Sal. typhimurium* (PDB ID: 1R2F), and RNR beta chain from *E. coli* (PDB ID: 1XIK) from the RCSB Protein Data Bank. Homologous sequences were then identified using psi‐blast 48 searches of the nonredundant protein database at the National Center for Biotechnology Information (NCBI). 472 related HO, thiaminase, MMOH, and RNR amino acid sequences were collected with percentage identities ranging from 99% to 4%. These sequences were initially aligned using t‐coffee 49. The alignment was manually adjusted using tertiary structure comparison of all structures using mapsci (http://www.geom-comp.umn.edu/mapsci/) 24 and through the RCSB PDB Protein Comparison Tool‐jFATCAT method 50, 51 using pairs of structures as a guide. The alignment editor used was genedoc 52. Tertiary structures were used to assess the structural or functional roles of conserved residues within the alignment. Molecular visualization was performed using rasmol 53. Analysis of conserved sequence motifs was facilitated by meme program, and these motifs were searched against a protein database using mast 36. Group entropy analysis (GEnt ) 40 was performed on the entire alignment to compare HO, MMOH, RNR, and thiaminase groups to each other. Evolutionary trace (http://mordred.bioc.cam.ac.uk/~jiye/evoltrace/evoltrace.html) 45, 46 was also performed on the entire alignment. Protein residue conservation prediction (http://compbio.cs.princeton.edu/conservation/score.html) 47 was performed on subalignments of HO, MMOH, RNR, and thiaminase groups. Each algorithm was used using combinations of all three possible backgrounds (BLOSUM62, SwissProt, and PF) and all seven possible matrices (BLOSUM62, BLOSUM35, BLOSUM40, BLOSUM45, BLOSUM50, BLOSUM80, and BLOSUM100) distributed with the program.

The phylip suite of programs was used to generate phylogenetic trees 54. First, the alignment was trimmed using trimal 55. 250 bootstrapped data sets of the trimmed alignment were then generated using the seqboot program. Next, evolutionary distances for each data set were determined by the protdist program using the Jones–Taylor–Thornton matrix. Phylogenetic trees for each data set were generated using the neighbor program. Lastly, the unrooted consensus tree was generated using the consense program. The tree graphic was generated using figtree (available at http://tree.bio.ed.ac.uk/software/figtree). A parsimony tree was generated using 50 bootstrapped data sets using the protpars program, followed by consense.

## Author contributions

JI performed the alignment of 472 HO homologue sequences, analyzed the alignment for functional, structural, and phylogenetic conservations, and drafted the manuscript. AR performed functional residue prediction analyses and interpreted the results. JP supervised the project, participated in the analysis of the alignment, helped to write the initial and final drafts of the manuscript, and addressed reviewer's comments.

## Supporting information


**Fig. S1.** Complete alignment of 472 heme oxygenase homologue sequences (MSF format).

**Fig. S2.** Unrooted bootstrapped parsimony tree of the heme oxygenase homologues. Branches are color‐coded based on enzyme type: blue = HOs, purple = thiaminases, red = MMOHs and green = RNRs. Specific subgroups within each enzyme group are labeled.

**Table S1.** Complete GEnt results of HOs.

**Table S2.** Complete GEnt results of thiaminases.

**Table S3.** Complete GEnt results of MMOHs.

**Table S4.** Complete GEnt results of RNRs.
