# Supplementary figures and images for: In silico analysis of heme oxygenase structural homologues identifies group‐specific conservations
Source: FEBS Open Bio. 2017 Sep 4;7(10):1480–98. doi: 10.1002/2211-5463.12275 (PMC5623701; doi:10.1002/2211-5463.12275)

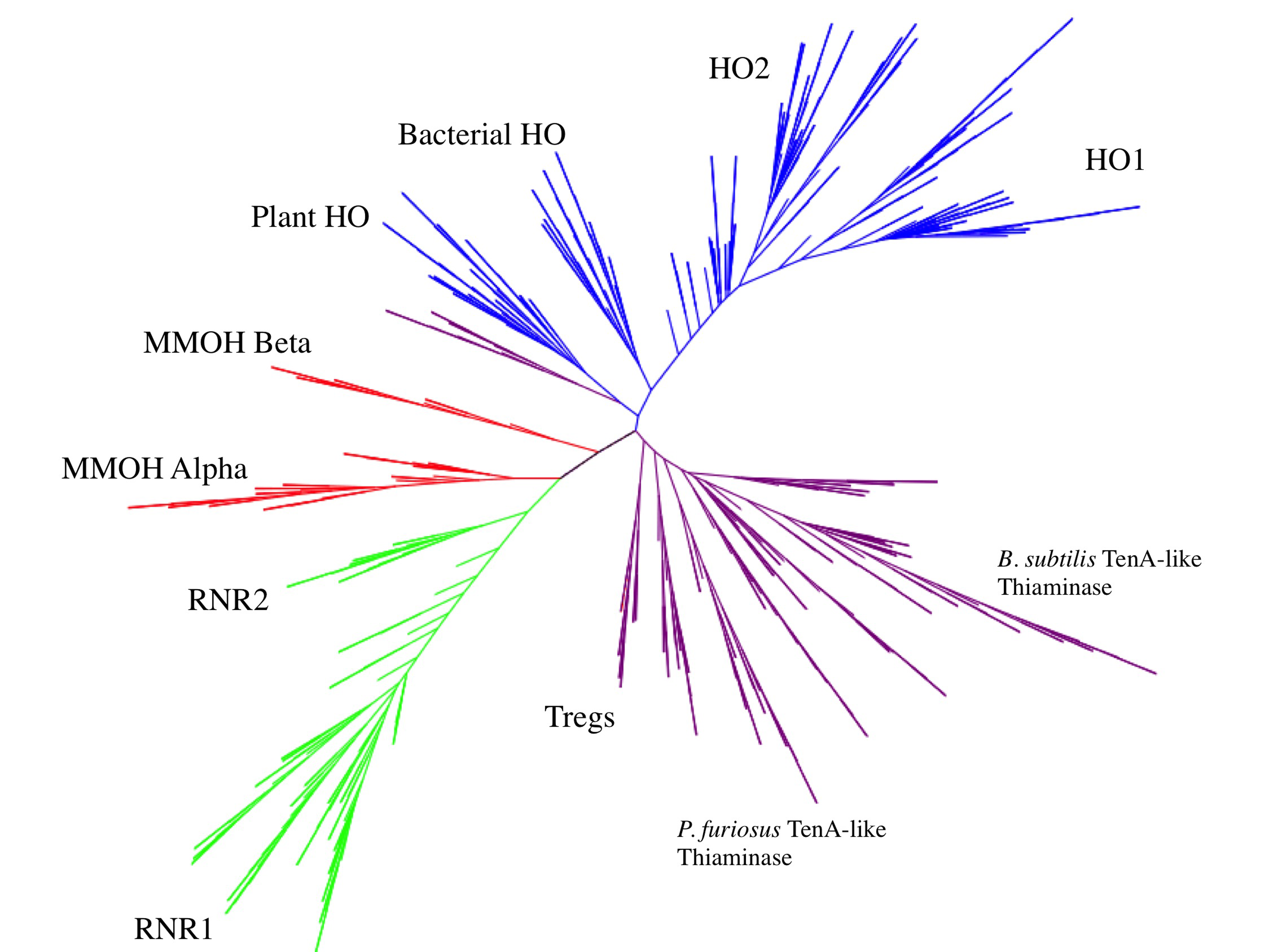

Supplement: Supplementary file 2 — Fig. S2. Unrooted bootstrapped parsimony tree of the heme oxygenase homologues. Branches are color‐coded based on enzyme type: blue = HOs, purple = thiaminases, red = MMOHs and green = RNRs. Specific subgroups within each enzyme group are labeled. [file FEB4-7-1480-s002.tif]
